# Individual Recognition in Domestic Cattle (*Bos taurus*): Evidence from 2D-Images of Heads from Different Breeds

**DOI:** 10.1371/journal.pone.0004441

**Published:** 2009-02-12

**Authors:** Marjorie Coulon, Bertrand L. Deputte, Yvan Heyman, Claude Baudoin

**Affiliations:** 1 Université Paris 13, CNRS UMR 7153, Laboratoire d'Ethologie Expérimentale et Comparée, Villetaneuse, France; 2 ENVA, Département Ethologie, Maisons-Alfort, France; 3 INRA, UMR 1198 Biologie du Développement et Reproduction, Jouy-en-Josas, France; University of St. Andrews, United Kingdom

## Abstract

**Background:**

In order to maintain cohesion of groups, social animals need to process social information efficiently. Visual individual recognition, which is distinguished from mere visual discrimination, has been studied in only few mammalian species. In addition, most previous studies used either a small number of subjects or a few various views as test stimuli. Dairy cattle, as a domestic species allow the testing of a good sample size and provide a large variety of test stimuli due to the morphological diversity of breeds. Hence cattle are a suitable model for studying individual visual recognition. This study demonstrates that cattle display visual individual recognition and shows the effect of both familiarity and coat diversity in discrimination.

**Methodology/Principal Findings:**

We tested whether 8 *Prim'Holstein* heifers could recognize 2D-images of heads of one cow (face, profiles, ¾ views) from those of other cows. Experiments were based on a simultaneous discrimination paradigm through instrumental conditioning using food rewards. In Experiment 1, all images represented familiar cows (belonging to the same social group) from the *Prim'Holstein* breed. In Experiments 2, 3 and 4, images were from unfamiliar (unknown) individuals either from the same breed or other breeds. All heifers displayed individual recognition of familiar and unfamiliar individuals from their own breed. Subjects reached criterion sooner when recognizing a familiar individual than when recognizing an unfamiliar one (Exp 1: 3.1±0.7 vs. Exp 2: 5.2±1.2 sessions; *Z* = 1.99, *N* = 8, *P* = 0.046). In addition almost all subjects recognized unknown individuals from different breeds, however with greater difficulty.

**Conclusions/Significance:**

Our results demonstrated that cattle have efficient individual recognition based on categorization capacities. Social familiarity improved their performance. The recognition of individuals with very different coat characteristics from the subjects was the most difficult task. These results call for studies exploring the mechanisms involved in face recognition allowing interspecies comparisons, including humans.

## Introduction

Individual recognition refers to a subset of recognition that occurs when one organism identifies another one according to its unique distinctive characteristics [1]. Individual recognition might be achieved through several sensory modalities. It is assumed that animals form mental representations of several common features of conspecifics as well as of unique individual sets of features of particular conspecifics [2]. For example in hamsters, *Mesocricetus auratus*, various odours correspond to various parts of body. A hamster which has previously interacted with another individual will treat all the odours from various parts of the body as belonging to that individual. However, a naïve hamster will associate each odour with a different individual [3]. Individual recognition is equivalent to a particular form of categorization phenomenon [4]. One individual constitutes a category in itself that includes all the different features of this distinct individual. Individual recognition might play an important role in social life, as animals which recognize the identity of a group member also recognize its species, its gender, its kinship, and its social status. Individual recognition has been demonstrated in invertebrates, in reptiles, in birds, in fishes, and in mammals (e.g. [5]–[9] respectively). Although individual recognition might be based on several sensory modalities in several species, animals might be able to recognize a congener using only one sensory modality. For example, emperor penguins, *Aptenodytes forsteri*, are similar morphologically but have a vocal signature that allows individual recognition [10].

The capacity of visual individual recognition has been studied in many invertebrate as well as vertebrate species (for reviews: [1], [11]). Among invertebrates, Tibbetts [12] demonstrated that wasps visually recognized an individual using head patterns. More recently, Van der Velden et al. [13] showed that crayfish can recognize an individual previously met during a fight and that this recognition was based on facial width or other facial features. In various vertebrate species, face recognition is the process the most commonly used to achieve visual individual recognition. Experimental studies in birds, sheep or primates (for review: [11]) used 2-D photographs of faces as stimuli. Visual individual recognition was demonstrated through two-step experiments in which animals were trained to discriminate slides of conspecifics and were then presented with novel slides of the same conspecifics in transfer tests. For example, Parr et al. [14] showed that rhesus monkeys and chimpanzees recognize unfamiliar individuals using facial cues from digitized static images.

Electrophysiological studies provided additional indirect evidence of individual recognition. Thus in macaques and sheep, neuronal circuits in the temporal cortex responded preferentially to faces in contrast to other visual stimuli (e.g. [15], [16] respectively). In the brain of sheep, separate face sensitive populations of cells are either view-dependant or view-independent [17]. View-dependent cells are used primarily in accurate and rapid identification of faces. View-independent cells are implied in recognition processes. In sheep, Peirce et al. found a right brain hemisphere advantage in the recognition of familiar faces of conspecifics [18] but not in the recognition of human faces [19].

When using 2D-images, to assert a genuine capacity for individual visual recognition rather than pattern recognition, the stimuli should include both representations of unfamiliar individuals and those of familiar conspecifics living in the group of the subjects [4]. Stable relationships between social group members have likely facilitated individual visual recognition. If this facilitation exists we can assume that subjects treat the slides as representations of real animals [4]. Dasser [20] recorded the responses of two female long-tailed macaques and showed that these females recognized novel pictures (in transfer test) only if the pictures represented familiar group members. In sheep, Kendrick [21] showed that social familiarity improved the animals' ability to discriminate between the faces (frontal and profile views) of individuals from a familiar breed. Moreover Ryan and Lea [22] showed that hens, in a transfer test, performed better when they were presented with novel slides of familiar rather than unfamiliar hens.

So it seems that for social species it is easier to recognize familiar rather than unfamiliar faces. However, there are few studies evaluating the capacity of categorization of various representations of an individual within the context of visual individual recognition [4] which would require discrimination of individuals within the set of familiar individuals.

Cattle (*Bos taurus*) is a good candidate species for addressing these questions.

*Bos taurus* is as a domestic species which allows the testing of several subjects (with *Prim'Holstein*, the dairy breed the most represented). In addition, cattle provide a great intra and inter-breed variety of coat characteristics (colour and patterns) and morphological diversity. Hence cattle are a good model for studying individual visual recognition. In addition cattle have good capacities of visual discrimination and they are able to discriminate between live familiar conspecifics [23]. Also we previously showed that cattle can visually discriminate 2D-images of their own species from other domestic species [24]. There is clear evidence that cattle can use vision to recognize other cattle [25]–[28] but direct behavioural evidence that cattle can visually recognize other cattle is still lacking. The aim of this study was to evaluate visual individual recognition capacities in cattle. Responses were obtained through an instrumental conditioning using images of faces of individuals as discriminative stimuli with food as positive reward. Individual recognition would be assumed by asking: do subjects treat different face views of the same stimulus animal as equivalent and is there a difference of performance depending on the various orientations of the face? In Experiment 1, all images represented familiar cows (belonging to the same social group) from *Prim'Holstein* breed (PH). In Experiments 2, 3 and 4, images were from unfamiliar (unknown) individuals either from the same breed or other breeds (*Normande* N and *Charolaise* CH breeds). These breeds are respectively characterized by similar coat patterns as PH subjects but with a different colour (brown N instead of black PH) or a uniform white coat colour without any spotted pattern (CH). In the present study all heifers recognized familiar and unfamiliar individuals from their own breed. In addition almost all subjects recognized unknown individuals from different breeds, however with greater difficulty.

## Results

**Figure 1:**
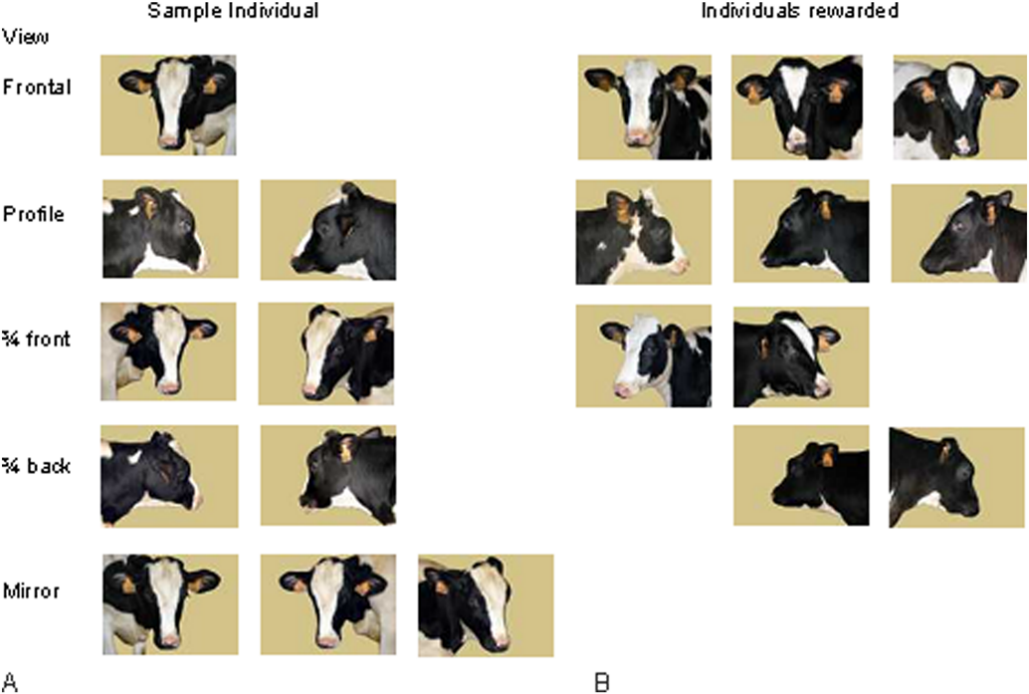
Example of stimuli used in the experiment of the recognition of a familiar *Prim'Holstein* individual. Ten views represented the sample individuals (A) and ten views represented three other individuals (B). In training, a frontal view of a face (the first line of the figure) of the sample individual (A) had to be discriminated from a frontal view of an individual in the group (B). In generalization test, for each trial, an image of the sample individual (A) and an image of a cow from the group (B) were randomly selected and presented simultaneously. For the second experiment, individuals were unfamiliar *Prim'Holstein* cows, for the third experiment unfamiliar *Normande* cows and for the last experiment unfamiliar *Charolaise* cows.

**Figure 2:**
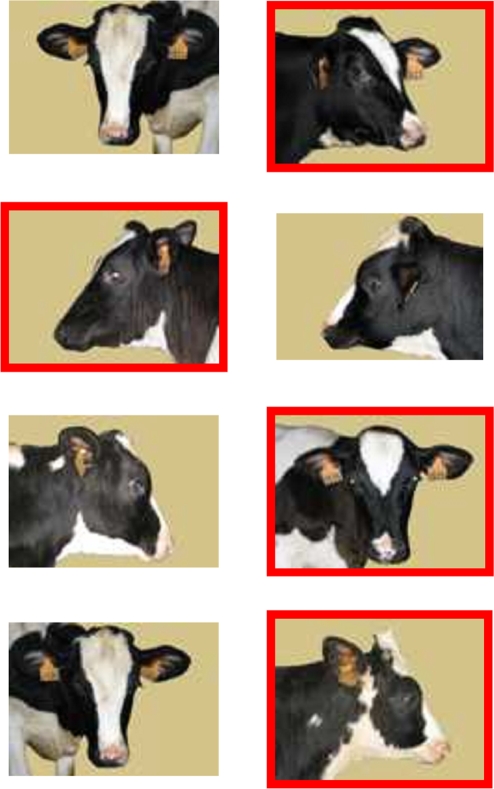
Individual recognition of a familiar *Prim'Holstein* cow. Example of the pairs of stimuli presented in consecutive trials of the generalization test. The stimulus rewarded is framed in red.

Eight PH heifers were tested with an instrumental conditioning with food reward. They were individually introduced into a test pen which included a central lane made of rows of straw bundle. A guillotine gate ended the central lane from which position heifers could see a pair of stimuli. When the subject had observed both stimuli, the experimenter lifted the gate. Then the heifer walked to the chosen image and accessed a reward placed behind an opaque panel (the test pen and procedures were described in more details in [24]). A test session included ten consecutive trials. Stimuli were photographs of head of heifers under various angles (frontal, profile, ¾ front views, ¾ back views and mirror views, Figure 1) on the same yellowish background. In the training phase, the same pair of stimuli was used in each trial. This pair was composed of a front view of the heifer to identify ( = sample individual) with a front view of another heifer (Figure 1). The subject had to choose the image of the other heifer to access to the reward. In generalization test, we used new photographs of the sample heifers and of other heifers, and the pair of stimuli was changed on each trial (Figure 2). The subject always had to choose the image of the other heifer to be rewarded. The criterion of success was for the heifer to make at least 8 correct choices per session in two consecutive sessions of 10 trials. The eight heifers were tested in four different experiments each of which made of a training phase and a generalization test. In the first experiment, we used images of familiar *Prim'Holstein* (FPH) heifers, in the second experiment images of unfamiliar *Prim'Holstein* (UPH) heifers, in the third experiment images of unfamiliar *Normande* (N) heifers and in the fourth experiment images of unfamiliar *Charolaise* (CH) heifers. Experiments 1 (FPH) and 2 (UPH), on the one hand, and experiments 3 (N) and 4 (CH), on the other hand, were paired. In each pair of experiments, half of the subjects were assigned to one experiment to start with (1 or 2 and 3 or 4). This procedure was intended to avoid a “carry-over” effect.

### Performances in experiments of individual recognition

**Figure 3:**
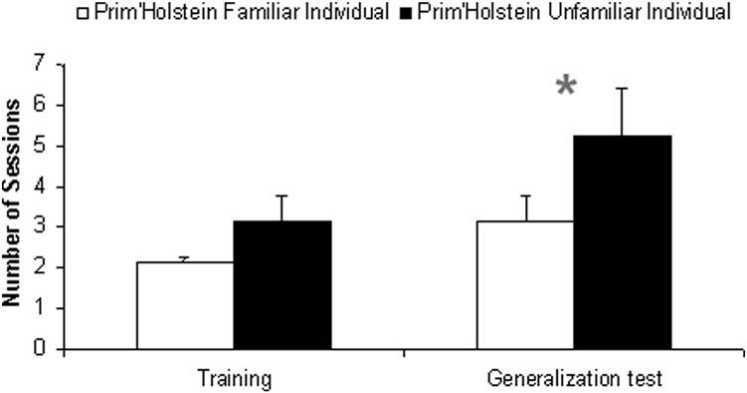
Individual recognition of a familiar versus an unfamiliar conspecific. Number of sessions (Mean+SE) to reach the criterion level during the training and the generalization phases in experiments 1 (familiar *Prim'Holstein*) and 2 (unfamiliar *Prim'Holstein*) (N = 9, * = P<0.05).

**Figure 4:**
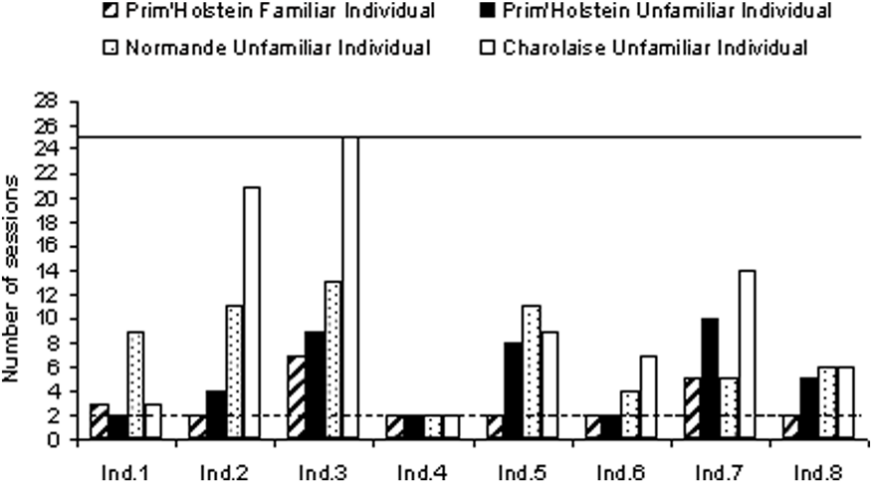
Performance of heifers during the generalization tests of the four experiments. Subjects recognized a familiar *Prim'Holstein* individual (experiment 1), an unfamiliar *Prim'Holstein* individual (experiment 2), an unfamiliar *Normande* individual (experiment 3) and an unfamiliar *Charolaise* individual (experiment 4). The minimum number of sessions to validate the criterion level (8/10 in two consecutive sessions) is indicated with a dotted line and the maximum number of sessions realized in an experiment (25 sessions) with a continuous line. One session corresponds to 10 consecutive trials. Along the x axis, subjects (Ind. 1 to Ind. 8) are sorted according to decreasing age (oldest to youngest).

**Figure 5:**
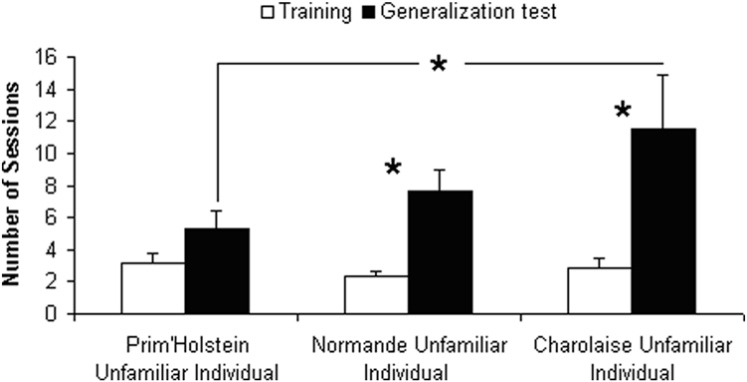
Individual recognition of an unfamiliar individual of the same *Prim'Holstein* breed and of other breeds. Number of sessions (Mean+S.E.) to reach the criterion level during the training and the generalization phases for the experiments 2 (*Prim'Holstein* breed), 3 (*Normande* breed) and 4 (*Charolaise* breed) (N = 9, * = P<0.05, ** = P<0.01).

In all experiments, whatever the sample individual (conditions: FPH, UPH, N, CH), all eight subjects successfully reached the criterion in the training phases. In generalization tests, all subjects recognized a familiar as well as an unfamiliar PH individual and an unfamiliar N individual (Figures 3, 4, 5). In addition all heifer subjects, except one recognized the unfamiliar CH cow from the other unfamiliar CH ones (Figures 4, 5).

### Comparison between training and generalization test

In the recognition task of a PH cow, performances of subjects during the generalization test did not differ from those of the training tests (*Z* = 0, *N* = 8, NS and *Z* = 1.36, *N* = 8, NS, Figure 3). For the first two sessions of the generalization test and for each subject, the number of errors was lower than expected by chance, between 0 and 7 errors across the 20 trials. However the ability to show recognition of the unfamiliar individual (breeds N and CH) tested against other (N and CH) unfamiliar individuals required significantly more trials during the generalization phase than during the training phase (*Z* = 2.37, *N* = 8, *P* = 0.018 and *Z* = 2.1, *N* = 8, *P* = 0.036, Figure 5). Moreover in experiments 3 (N), and 4 (CH), two and one subjects respectively performed more errors than expected by chance in the first two sessions of the generalization test.

### Is it easier for a heifer to recognize a group mate than an unknown individual?

In the first experiment stimuli, referred as to “familiar”, featured individuals were group mates of the subjects. In the second experiment all stimuli displayed individuals of the same breed that were unknown to the subjects (“unfamiliar”). On average, subjects reached criterion sooner when recognizing a familiar PH individual than when recognizing an unfamiliar one (FPH vs. UPH; Generalization tests: Exp 1 vs. Exp 2; *Z* = 1.99, *N* = 8, *P* = 0.046, Figure 3). Training performances were similar.

### Is it easier for PH heifers to recognize an unknown individual with a spotted coat (similar to their own coat; UPH vs. N) than an unknown individual with a uniform white coat (different from that of the subjects; UPH vs. CH)?

During the training phase, the discrimination of two unfamiliar individuals did not differ whatever their coat was similar or different to that of the subjects (UPH vs. N; *Z* = 1.6, *N* = 8, NS and UPH vs. CH; *Z* = 0.18, *N* = 8, NS, Figure 5).

During the generalization phase, heifers had more difficulties recognizing unfamiliar individuals with a uniform coat (CH) than others unfamiliar heifers of their own breed (CH vs. UPH; Generalization tests: Exp 2 vs. Exp 4; *Z* = 2.37, *N* = 8, *P* = 0.018, Figure 5). In contrast recognizing an unfamiliar N individual (spotted coat) was as easy as recognizing an unfamiliar PH (N vs. UPH; Generalization tests: Exp 2 vs. Exp 3; *Z* = 1.52, *N* = 8, NS, Figure 5).

### Temporal variables

The previous results dealt only with successes in choosing the rewarded stimulus. However temporal variables of subjects during each trial were also recorded: the “Time to the Gate” (TG, the time spent to get to the guillotine gate) and the time elapsed from the lifting of the gate to the arrival at the selected image. This latter time variable seemed to be a potentially good measure of the difficulty of heifers in making their choices, but the means remained similar in our experiments. However TG, which seemed to be a good measure of how motivated the heifers were to carry out a trial, was greater when subjects were presented with two CH stimuli (uniform white coat) than when presented with two PH ones (Trainings: TG_UPH_ vs. TG_CH_; *Z* = 2.2, *N* = 7, *P* = 0.028; Generalization tests: TG_UPH_ vs. TG_CH_; *Z* = 2.52, *N* = 8, *P* = 0.012, Table 1).

**Table 1:** Influence of categories of stimuli on the time subjects spent reach the gate from the back of the experimental room (Time to the Gate, TG).

		TIME TO THE GATE (TG, sec)
Familiar *Prim'Holstein*	Training	2.38±0.12
	Generalization test	2.33±0.11
Unfamiliar *Prim'Holstein*	Training	2.36±0.24
	Generalization test	2.35±0.16
Unfamiliar *Normande*	Training	2.79±0.15
	Generalization test	2.91±0.15
Unfamiliar *Charolaise*	Training	2.77±0.19
	Generalization test	2.92±0.17

### Is the recognition process affected by the orientation of the face?

For each experiment and possible pair of view angle of stimuli, we calculated the error rate (ER). The ER did not vary with the angle view of the sample individual (*F*_4,464_ = 1.38, NS) but changed differentially with the view angle of the two stimuli in the four experiments. These differences arose from the nature of the stimuli (FPH, UPH, N and CH) interacting with the different view angles (General linear model ANOVA: experiment×view angle of sample individual×view angle of others individuals: *F*_36,464_ = 1.58; *P* = 0.02). However, the ER did not differ whether the view angles of the stimuli were the same or different (ER_same_ = 0.25±0.03 vs. ER_different_ = 0.24±0.02, *U* = 340, N_1_ = 16, N_2_ = 47, NS).

## Discussion

These results demonstrated efficient visual individual recognition in cattle achieved using 2D-images. Heifers treated equivalently all views of the head of one individual whether or not they had previously interacted with it. However social familiarity improved their performance. This confirms that cattle treated the 2D images as genuine representations of conspecifics and not as an arbitrary visual object. The more different the coat pattern was, compared to that of the subject, the more difficult was the recognition.

Subjects, trained to discriminate frontal views of two individuals, can recognize new views of the individual to be recognized compared to other individuals. This capacity was also demonstrated with unknown individuals and with individuals with a different coat colour or pattern, without prior interaction with the individuals to be recognized. Our results extend the cognitive capacities in cattle that we previously demonstrated for visual discrimination of species [24]. The cognitive capacities of visual individual recognition in cattle match those shown in other species like birds [22], [29], sheep [21], macaques [20], rhesus monkeys and chimpanzees [14]. Contrary to studies in other species, we used five different view angles (frontal view, profile views, ¾ front views, ¾ back views, mirror views) of the individual to be recognized combined with both right and left orientations, so a total of ten different orientations. In spotted breeds like *Prim'Holstein* or *Normande* cows, the right side is different from the left side. In sheep, Kendrick [30] showed that facial discrimination occurred with two face orientations (frontal and profile views). Ferreira et al. [31] showed, in the same species, that the learned discrimination of the profile views of a pair of unfamiliar adult faces did not improve subsequent discrimination of frontal views of the same pair. In our study heifers grouped all the different views of the same individual into a similar category. According to Zayan and Vauclair [4], these results may indicate that the polymorphous set of each known individual's features becomes mentally represented as a single natural category, comprising the morphological properties unique to each animal. However our results could also be interpreted as referring to a process of mental rotation of images. Based on this hypothesis, we may expect that the performance recorded in the generalization test (exp. 1 and 2) would be similar in the recognition of familiar and unfamiliar individuals. This prediction was not verified, indicating that a true social recognition occurs rather than a simple mental rotation process. The capacity of individual recognition extends to unknown individuals. This suggests that the subject formed an integrated representation of each conspecific built up from the set of specific features of an individual. Consequently cows might very rapidly be able to recognize new social partners.

The recognition process of a familiar individual was rather straightforward. This suggests that whatever a partner's physical orientation, a cow is able to maintain its partner's identification process. This is in line with the stability of herds of cattle [27]. The heifers recognized more easily a familiar individual than an unfamiliar one. Individual recognition was very likely facilitated by information acquired through recurrent social interactions within the group. In sheep, Kendrick et al. [21] showed that faces of socially familiar animals enhanced significantly the speed of learning the recognition task and in macaques, Dasser [20] demonstrated that the animals correctly matched facial views with novel slides of other body parts of the same animal only if that animal was a familiar group member. Kendrick et al. [32] showed also that there was a progressive increase in the number of cells in the brain which selectively encoded faces of members becoming familiar. Domestic cocks [33] and hens [22] recognized a familiar individual as easily as an unfamiliar one. These animals can discriminate the individuals as distinct categories but not necessarily as individual conspecifics. The fact that heifers had more difficulty to recognize unfamiliar individuals than familiar ones suggests that they likely consider 2D-images of conspecifics as genuine representations of live animals as Zayan and Vauclair asserted [4]. This assumption is supported by emotional responses observed during trials. Our subjects held their ears backward ten times more when processing images of unfamiliar individuals than when processing stimuli from familiar individuals. Ear postures were shown to be emotional cues in several mammalian species (horse [34], sheep (Désiré, 2006, unpublished data), dog [35] or wild canids [36]) while a backward position indicating a “negative anticipation”[37]. In addition, a short time spent to get to the guillotine gate could be interpreted as the high motivation of the heifers. Our subjects spent more time to go to the gate when processing recognition task of unfamiliar *Charolaise* images. These stimuli proved to be the most difficult to process by our subjects and lead to the greatest level of frustration (no reward). Therefore success rates, ear postures and readiness to perform the task all concur to confirm that our subjects were treating images as representations of live conspecifics.

We showed that the capacity of visual recognition of an unfamiliar individual could be extended even to individuals from a different breed with a different coat pattern. The heifers recognized individuals from *Normande* and *Charolaise* breeds, but this recognition was more difficult. Based on the reduced number of errors in the first sessions we can consider that a real generalization process was involved in the recognition of a cow of the same breed. In the recognition of a *Normande* or a *Charolaise* cow our results suggest a different mechanism. The lowest performances, compared to the previous ones, could be interpreted by the necessity to learn the different images corresponding to a particular individual. Whereas all heifers succeed in recognizing a *Normande* individual, the coat of which presenting some similarity to their own coat (spotted coat), with only one subject failing to reach the recognition criterion (after 250 trials) when presented with a *Charolaise* individual, the coat of which being of a uniform white colour, with no spots whatsoever. As it has been described in goats for mother recognition by kids [38], in cows it seems likely that coat markings or spots are important features for the recognition process. When chickens from different breeds interact, it seems that they performed breed rather than individual recognition [39]. In contrast, cows are able to perform individual recognition independently of the breed to which individuals belong. However subjects seemed disoriented when they had to base their recognition on features different from those they previously relied on. A same effect has been shown in humans who have more difficulties in recognizing dissimilar individual faces from different ethnic groups [40].

In our study the error rate changed accordingly when two stimuli in the discrimination task were under different orientations, and this effect was based on the breeds shown in the stimuli. However the error rate did not vary with the angle view of the sample individual. Our results are in agreement with those of Sato and Yoshikawa [26], who showed that their two cow subjects attended the same amount of time to the frontal, profile and back views of a cow stimulus. These results contrast with those of Bruce et al. [41] who showed, in humans, that the recognition of an unfamiliar individual was the easiest with ¾ frontal views and the most difficult with profile views. Perhaps, other features like attributes of faces (e.g. eyes, internal or external features) can have an influence on the performances in individual recognition. Configural cues are important in chimpanzee face processing [42] and Parr et al. [14] showed that the eyes were the most important cues for individual recognition in chimpanzees and macaques. In sheep Peirce et al. [18] showed that only familiar faces could be recognized using the internal features alone. External features could be used to identify both familiar and unfamiliar faces. Key facial features like ear position and appearance of the eyes were used for processing recognition of emotions in sheep [17]. Peirce and Kendrick [43] suggested that nerve cells in the right hemisphere may play a key role in the rapid identification of facial identity. Cells in the left hemisphere may be more specifically involved in slower processes associated with facial emotion recognition. Face-based emotion recognition was shown also in chimpanzees [44], [45] depending on several key features such as the shape of eyes or mouth. The sustained rather high rates of success in our experimental heifers suggest that social stimuli presented as 2D-images were appropriate stimuli facilitating picture recognition [11].

In conclusion we have demonstrated that cows are capable of visual individual recognition in discriminating within a similar category different views of one individual. This especially applies to familiar individuals, though this capacity also extends to unfamiliar individuals from the same or different breeds as the subjects. However the individual recognition task proved to be the most difficult when the visual features of the breed being tested were quite different, as with no spots, from that of the subject (spotted breed).

## Materials and Methods

### Subjects

Eight *Prim'Holstein* heifers participated in this study. They were produced by artificial insemination. All were born at the UCEA INRA experimental farm in Bressonvilliers (France). The subjects ranged from nine to 14 months of age and weighed a mean of 277±13 kg at the beginning of the study. All animals were housed in the same nursery in individual box stalls in similar conditions from birth until 6 months of age. Afterwards they were grouped together in the same loose housing system (11×18 m) with 16 other heifers. These latter heifers were age matched to the subjects. All animals had free access to water. They were fed the same standard diet (grass silage, hay, corn straw and mineral). All subjects had been extensively trained in the discrimination procedure for several months. All animals lived under natural and/or artificial light according to the season. In the latter case light was on between 6:00 and 19:00. Each cow was identified with an I.D. number printed on two ear tags.

### Procedure

#### Stimuli

The stimuli consisted of 80 prints (42×50 cm) of digitised colour pictures with 20 prints of four *Prim'Holstein* heifers socially familiar to the subjects, 20 prints of four *Prim'Holstein* heifers unfamiliar to the subjects, 20 prints of four *Normande* unfamiliar heifers and 20 prints of four *Charolaise* unfamiliar heifers. Each set of 20 prints was utilized in one experiment (FPH, UPH, N or CH) and represented faces of heifers from different view angles (frontal views, right and left profile views, right and left ¾ frontal views, right and left ¾ back views and mirror frontal views). In each experiment, the 20 views consisted of 10 different views of the same individual to be recognized (sample individual) and 10 views of three other individuals (Figure 1). In each category, the 10 stimuli were sorted randomly from a larger set of stimuli. All the stimuli were presented at approximately the natural size of the head of a cow. The original background of all pictures was replaced by the same uniform background, a yellow colour mimicking that of straw (D2C48A background, Adobe Photoshop Elements ©).

#### Apparatus

The paradigm was based on a simultaneous discrimination of S+ and S− stimuli. Responses were obtained by means of instrumental conditioning using positive food rewards. The instrumental conditioning apparatus was placed in a test pen (6×11 m) adjacent to the free stall where the group of subjects lived. Subjects were tested individually while remaining in auditory contact with their group members of the adjacent free stall. The instrumental conditioning and test pen were the same as described in Coulon et al. [24]. In the test pen, the subject walked to a guillotine gate at the end of a lane made between rows of straw bundles. From the gate the heifer could see the two images placed at its eye level. For each pair of stimuli, one stimulus was consistently associated with a reward, S+. After the heifer has looked at both stimuli, the experimenter lifted the gate from behind the subject. In every case the experimenter waited at least 5 sec after that the heifer arrived at the guillotine gate before lifting it. The heifer could then walk towards the chosen image. On the correct side (S+ side), the opaque panel could be pushed by the subject to get access to the reward. On the incorrect side (S− side), the panel was blocked. To avoid any olfactory bias a reward was always placed behind each panel. The left/right position of the rewarded stimulus was randomly balanced across trials.

#### Protocol

The instrumental conditioning procedures were similar to those described by Coulon et al. [24]. Each session consisted of 10 trials. For each subject, two successive sessions were completed in the morning (between 8:00 and 12:00). At least 48 hours elapsed between each block of two sessions. Before being tested in these experiments, the heifers went through a phase of habituation to the apparatus and had participated in experiments using the same device. Each experiment included a training test and a generalization test.

***Training***. One photograph, a frontal view of the head of the individual to be recognized, S−, and one photograph in frontal view of a different individual, S+, were used (Figure 1). Each session consisted of 10 trials with the same pair of stimuli. The criterion for success in the training phase was at least 8 correct choices per session in two consecutive sessions of 10 trials each (*P* = 0.01).

#### Generalization test

Eighteen new stimuli were introduced in generalization test sets, therefore all 20 stimuli were used (Figure 1). The pair of stimuli - one picture of the individual to recognize (S−) in a specific view angle and one photograph of another individual (S+) in the same view or in another view - was changed at each trial (Figure 2). The pair of stimuli was drawn randomly from the entire set of 100 pairs. The same stimulus was not presented in more than three consecutive trials. Each session consisted of 10 trials and the heifer had to avoid choosing the image of the individual to be recognized to make a correct choice and to receive a reward (Figure 2). The criterion of success in the generalization phase of experiment 1 was the same as the training phase.

#### Control trials

Parallel studies were completed to control that there was not effect of the experimenter and the relevance of stimuli. First studies showed that the subjects chose the image that they observed the most and not the last stimuli looked at before the gate was lifted. It was clear that the experimenter did not influence the choice of heifers. In another parallel control study, we had presented images of faces upside-down or faces in which only the eyes and the nose were visible. In both cases, the performances did not differ from chance and the error rates of subjects were 50%. These results showed a contrast to the relevance of our experimental stimuli (normal head images).

### Temporal variables

For each trial of each experiment, we determined from the video recording the “Time to the Gate” (TG, the time spent to get to the guillotine gate, in seconds) and the time elapsed from the lifting of the gate to the arrival at the chosen image.

### Error rates of view angle of stimulus face

For each subject, experiment and possible pair of view angle of stimuli, we calculated the error rate (ER = the number of errors divided by the number of errors plus the number of successes).

### Data Analysis

The primary variable was the number of sessions needed to reach the criterion. Due to small sample size and non-normally distributed data only non-parametric statistics were used. The Wilcoxon Signed-ranks test, *Z* statistic, was used to compare between experiments (1 versus 2, 2 versus 3 and 2 versus 4) for both the number of sessions to criterion and the two time variables. A general linear model ANOVA was used to analyse the influence of the experiment, the orientation of the face of the sample individual and that of the other individual on the error rate. We compared the error rate whether the orientations of the faces were the same or different using a Man Whitney *U* test. A two-tailed probability level *P* of 0.05 was used all throughout (NS: P>0.05). All means were presented with their standard errors (mean±SE). All analyses were performed with the statistical package Statistica®.

### Ethical Note

Animal care and all procedures were completed in accordance with the authorization B91 332 101 and 93-031 of the French Ministry of Agriculture and the EU directive. The protocol, registered as “protocol 06-002”, was approved by the Regional Ethical Committee of Paris-Sud.
